# Massive Screening of Food Extracts for Quality Assessment and Standardization of Allergenic Activity

**DOI:** 10.3390/bios14120615

**Published:** 2024-12-13

**Authors:** Luis Antonio Tortajada-Genaro

**Affiliations:** 1 Instituto Interuniversitario de Investigación de Reconocimiento Molecular y Desarrollo Tecnológico (IDM), Universitat Politècnica de València, Universitat de València, Camino de Vera s/n, E46022 Valencia, Spain; luitorge@qim.upv.es ; Tel.: +34-963877000; 2 Departamento de Química, Universitat Politècnica de València, Camino de Vera s/n, E46022 Valencia, Spain; 3 Unidad Mixta UPV-La Fe, Nanomedicine and Sensors, Instituto de Investigación Sanitaria La Fe, E46026 Valencia, Spain

**Keywords:** biosensing for quality control, food-induced allergy, allergen extracts, biotech and pharmaceutical industry, optical sensing

## Abstract

(1) Background: In drug discovery and pharmaceutical quality control, a challenge is to assess protein extracts used for allergy therapy and in vivo diagnosis, such as prick tests. Indeed, there are significant differences between the features of marketed products due to variations in raw materials, purification processes, and formulation techniques. (2) Methods: A protein array technology has been developed to provide comprehensive information on protein–biomarker interactions on a large scale to support the pharmaceutical industry and clinical research. The biosensing method is based on immobilizing low volumes of protein extracts (40 nL) on thermoplastic chips in array format. The biological activity was estimated by incubating with serum from representative food allergy patients. (3) Results: The reproducible optical signals were registered (deviation lower than 10%) using low-cost technologies such as a smartphone and a reader of digital versatile discs. The method was applied to pharmaceutical products to diagnose ten common food allergies, including barley, kiwi, milk, prawn, egg, peanut, wheat, peach, walnut, and squid. Quality indicators were established from spot intensities, enabling an effective comparison of manufacturers. (4) Conclusions: A biosensing-based strategy for screening pharmaceutical products emerges as a reliable and advantageous alternative to traditional approaches such as electrophoresis, fluorescence chips, and ELISA assays. This high-throughput method can contribute to understanding complex biological processes and evaluate the performance of pharmaceutical products.

## 1. Introduction

Data from bioanalytical methods represent one of the most novel aspects of pharmaceutical product development. A relevant example is methods for high-throughput screening in drug discovery [1]. Due to significant advancements in automation and miniaturization, even compound libraries can now be screened against numerous biological targets within a relatively short timeframe and at low costs compared to previous efforts. Thus, recent advances in biorecognition events and biosensing devices provide new approaches for drug screening, drug mechanism elucidation, and the characterization of target interactions on a large scale [2]. Another high-demand area is active testing to assess the quality of products generated at various stages of the manufacturing process [3]. This need is particularly significant when dealing with products of natural origin, such as protein extracts used in allergy diagnosis [4].

Worldwide, over 220 million people suffer from some form of food allergy. Nowadays, this disease is regarded as a public-health-relevant problem because the percentage of the affected population is high (5–10%) and is more common in children than adults [5]. A reliable diagnosis is essential to prevent adverse reactions related to allergens [6]. Skin and serum tests are indispensable in assessing patients with a suggestive clinical history of food allergy [7]. In both approaches, food extracts are employed as reagents for the selective recognition of the most important biomarker, i.e., specific IgE. Additionally, protein extracts are used in therapy because they are administered via subcutaneous injections or sublingual tablets to induce immune tolerance and reduce allergic reactions [8]. However, there are significant differences between allergen extract products in the current market. These inconsistencies may lead to protein composition, potency, and biological activity disparities, directly impacting their performances. For instance, a poor extraction can reduce the accuracy of diagnostic tests, leading to underestimated allergic responses and even the reporting of false negatives. Therefore, such variations between protein extracts underline the need for standardized evaluation to ensure consistency and reliability across products, ultimately improving patient care.

Preparing protein extracts from raw food and the standardization of the resulting products are challenging for the pharmaceutical industry, considering the applicable regulatory requirements [9,10]. Quality assessment of extracted fractions plays a crucial role in guaranteeing a sensitive detection of specific IgE and the effectiveness of allergen-specific immunotherapy [11]. The challenges include the choice of source material and manufacturing protocols. Several studies have addressed this topic, providing valuable insights and guidelines for evaluating their potency, purity, and stability [12,13]. Another important conclusion is the need to standardize allergenic activity and establish reference materials and methods to ensure consistency across different manufacturers [14,15].

Several methods are employed to control the production steps, supporting the consistency and standardization required for clinical practice. SDS-PAGE (sodium dodecyl sulfate-polyacrylamide gel electrophoresis) and capillary electrophoresis (CE) are commonly employed for purity analysis [16]. These techniques ensure the absence of contaminants such as impurities, host cell proteins, or residual chemicals used during extraction. Advanced proteomic techniques help investigate the presence of cross-reactive allergens and non-allergenic components [17]. Bioassays, such as cell-based assays or animal models, are commonly employed to assess potency [18]. These assays measure the functional activity of the protein isolates, such as their ability to bind to a specific receptor or induce a desired biological response. Techniques such as enzyme-linked immunosorbent assay (ELISA) are commonly employed to quantify the main allergens present in foods and other materials [4,19,20]. Immunoassays help determine the potency and concentration of allergenic components, ensuring consistency across different batches. Nevertheless, new high-throughput approaches are required to quickly assess the quality of allergen extracts and to predict their diagnostic efficacy.

This study demonstrates that a novel array technology is an efficient massive screening platform for testing food extracts. The assay principle is that the in vitro biosensing of specific IgE levels obtained from representative serum provides a reliable approach to accurately measuring biological activity. Thus, proteins from pharmaceutical products are attached to a solid support in a spatially defined pattern. The selected material was polycarbonate, chosen over glass or polystyrene, offering a cost-effective solution for immobilizing proteins, nucleic acids, or sugars, aimed at applications in molecular diagnosis [21]. Other attractive characteristics are impact strength, rigidity, heat resistance, and optical transparency. Polycarbonate is also easily moldable for the low-cost production of microfluidic designs via injection molding, suitable for high-throughput manufacturing processes. Significantly, its hydrophobic nature minimizes the non-specific binding of sample components, enhancing the signal-to-noise ratios in biosensing assays [22].

On the other hand, we also propose alternative detectors to conventional chip readers to provide straightforward and cost-effective imaging solutions and enhance accessibility and practicality for real-time control in production processes. Thus, based on the excellent results for DNA arrays, a simple detection approach combines planar chips and smartphone imaging [23]. Furthermore, DVD-based assays have been developed for more sensitive readouts. This optoelectronic technology has succeeded in biomarker determinations in human fluids, such as SNPs and mutation identification [24]. Recently, this technology has been applied to the clinical diagnosis of hypersensitivity mediated by immunoglobulins [25]. The analytical performance was excellent, with high selectivity, good repeatability, fast response, low detection limits, low consumption of reagents and serum, reliable readout, and validated diagnosis. The results demonstrated its reliable multiplexing capability against target-to-target solutions, such as ELISA or Immunocap technology. Therefore, the current research hypothesized that this novel method has a potential application in the pharmaceutical sector. Developing a versatile platform for the high-throughput analysis of IgE binding capacity allows for the quick and easy standardization of allergen extracts and facilitates their quality control.

## 2. Materials and Methods

### 2.1. Protein Extracts

The proteins from food sources were provided by three manufacturers, coded as M1, M2, and M3 (Table S1). The protein extracts included barley (*Hordeum vulgare*), cow’s milk (*Bos taurus*), kiwi (*Actinidia deliciosa*), prawn (*Parapenaeus longirostris*), chicken egg (*Gallus gallus*), peach (*Prunus persica*), peanut (*Arachis hypogaea*), and wheat (*Triticum aestivum*).

### 2.2. Chip Preparation

Two platforms were tested: bulk digital versatile discs (DVDs, radius 12 cm, 1.2 mm thickness) and polycarbonate slides (PC, Makrolon GP clear 099, dimensions 7.5 cm × 2.5 cm, 0.4 mm thickness). Chips were first conditioned by gentle ethanol washing, water rinsing, and air drying. Food extracts at 40 ng/mL were diluted in a printing buffer composed of 50 mM carbonate buffer at pH 9.6 and 1% glycerol (*v*/*v*). Spotting was performed using a liquid dispenser robot (Biodot AD1500, Irvine, CA, USA), depositing 40 nL of probe solution at 25 °C and 80% relative humidity. In the DVD-based assay, each disc contained 20 arrays on the top layer. In the slide-based assay, each chip held 20 arrays. In both cases, probes were spotted in a 4 × 3 format, i.e., four replicate spots per manufacturer. The internal assay quality, including spots with control reagents against the serum IgE, was checked. The positive and negative controls were mouse anti-human IgE monoclonal antibody (Ingenasa-Eurofins, Madrid, Spain) and human serum albumin at 10 ng/µL (HSA, Sigma-Aldrich, St. Louis, MO), respectively. The distance between flanking spots was 1.5 mm, and the size of the spots was 500 ± 10 µm. Chips were incubated overnight at room temperature, promoting surface coating by passive adsorption on the plastic substrate. A blocking step was not required. The chips were stored in a sealed box at 4 °C until use. Experiments confirmed that the probes were active for at least 30 days.

### 2.3. Preparation of Patient Standard Solutions and Buffers

Serum samples were collected in red-top tubes (BD Diagnostics, Madrid, Spain) and incubated at room temperature for 60 min to induce clotting. After centrifuging at 2000 rpm for 15 min, serum was aliquoted into cryovials and stored at −80 °C until used. The study included a cohort of 30 food-allergic patients and 20 controls. All the individuals were characterized according to their clinical history, prick-test result, and the concentration of the specific IgE measured by ImmunoCAP. The participants were enrolled after providing written informed consent according to the protocols approved by the Ethics Review Board (Hospital Universitari i Politènic La Fe Valencia, Spain). A hundred standard solutions were prepared by mixing equal volumes of patients’ serum with a 0.5% Tween-20.

The immunoreagent buffer was phosphate-buffered saline (PBS) prepared at 0.8% NaCl, 0.02% KCl, 0.02% KH_2_PO_4_, 0.3% Na_2_HPO_4_, pH 7.5. The washing buffer was PBS with 0.05% Tween-20 (PBST).

### 2.4. Assay of IgE Reactivity

Each patient standard solution was dispensed on the array containing the extracts from the compared manufacturers immobilized on the disc surface. After incubation for 30 min at room temperature, the disc was washed with PBST and water and dried. Next, the polyclonal anti-human IgE antibody (pAb) conjugated to horseradish peroxidase (HRP) was employed (Dr. Fooke Laboratorien, Neuss, Germany). The reagent was prepared in PBS at the 1/800 dilution. After incubation for 30 min at room temperature, the disc was washed as before. Colorimetric staining consisted of direct substrate dispensation. The ready-to-use reagent was tetramethylbenzidine (ep(HS)TMB-mA, SDT GmbH, Kraichtal-Münzesheim, Germany). After a 10 min incubation, washing with deionized water stopped the reaction.

### 2.5. Detection and Image Processing

Smartphone reading. A smartphone (model P Smart, Huawei, Shenzhen, China) registered the microarray images, as described in reference [24] and Figure S1. The Image J software (NCBI) (https://imagej.net/ij/) was used for image analysis.

DVD reading. The reading device acquired surface images and performed the data analysis to quantify spot signals, as described in the reference [25]. Briefly, custom software served as the reader control-modified DVD drive (DVD GSA-H42N, LG electronics, Seoul, South Korea) (Figure S2). The scanning parameters were speed 8×, gain 25 dB, and processing diameter 250 mm (460 pixels per spot). In-home software providing automated data analysis generated digital signals for all spots in each array.

For both devices, experimental noise values were calculated as the standard deviation from 15 blank measurements (regions without probes). The optical detection threshold was inferred from the experimental concentration corresponding to signal-to-noise ratios equal to 3. Thus, the detection limit was estimated as the extract concentration that modified 10% of the background signal. Assay reproducibility was estimated from replicates (intra-chip, inter-chip, and inter-day), evaluating pixel number per spot and standard deviation of spot intensity. The optical signal was determined for extract evaluation by subtracting the local background signals from the mean spot intensity.

## 3. Results

### 3.1. Assay Principle

**Figure 1:**
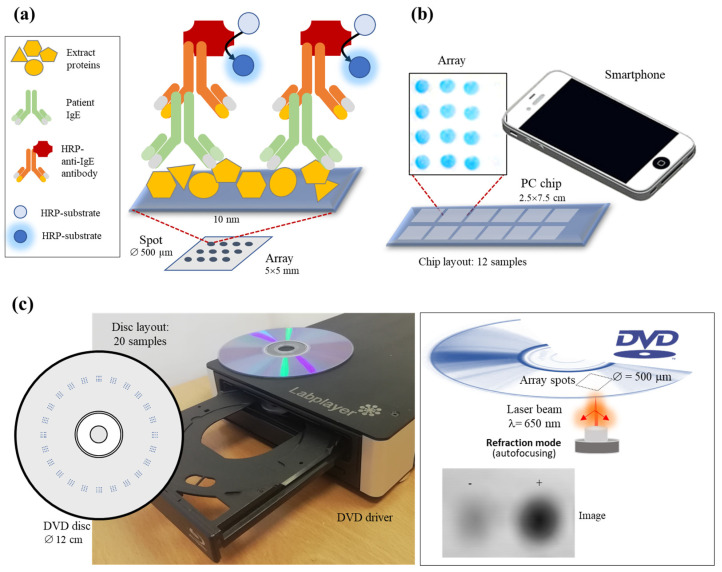
Principle of the assay: (**a**) Scheme of reverse-phase protein array. (**b**) Reading principle on a planar slide by smartphone camera under controlled illumination conditions. (**c**) Reading principle on disc surface by scanning the DVD laser beam. In reflection mode, the intensity of the reflected beam on the internal DVD layer changes in the presence of biorecognition elements on the disc surface.

The proposed method is supported by the principles of massive techniques based on protein arrays. In our approach, the quality of the food extract is related to the recognition between allergenic proteins and the associated immunoglobulin E produced in the human body (Figure 1a).

The initial step is immobilizing protein extracts containing the allergenic molecules of interest, using a low volume and a high-throughput mode. Later, food-specific IgE solutions obtained from representative serum samples from allergic patients are dispensed on the chip surface. Biorecognition mimics the typical response mechanism mediated by immunoglobulins. After an appropriate chip staining, an imaging system is used to register the array output. Two low-cost detectors were used instead of traditional fluorescence chip scanners: a smartphone (Figure 1b) and a DVD-based device (Figure 1c). Considering the demands of production control points, they offer a simple and accessible approach for analyzing various biological samples as screening tools. The data analysis of spot intensities will reveal a clear discrimination pattern depending on the extract quality.

### 3.2. Setup of Recognition Event

Multiple design considerations were considered for developing a cost-effective tool applied to large-scale protein extract evaluation. (i) The selected chip material was polycarbonate, a thermoplastic polymer known for its transparency, durability, and ease of fabrication. In addition, polycarbonate is a relatively inexpensive material, contributing to the cost-effectiveness of manufacturing microarray chips (Table S2). (ii) The proposed attachment of proteins onto the chip is direct immobilization without a sophisticated chemical modification of the surface and its hydrophobic nature that avoids an intensive blocking treatment. (iii) A straightforward method for detection was chosen, integrating planar chips with low-cost imaging technologies.

Initial tests were to confirm that the proposed technology was valid for a single protein–specific IgE interaction (Figure S3). The amount of immobilized allergen on the plastic chip or disc was critical for the analytical performance of the method, especially its sensitivity. Furthermore, the spot intensity was proportional to the recognition yield between the immobilized protein and the immunoglobulin. The biomarker concentration can be quantified using a given concentration of attached proteins, generating a diagnostic assay. However, by changing the target molecule, a quality assessment can be approached. The experiments demonstrated that the quantity of active proteins in an extract could be determined using a standard with a fixed and known amount of specific IgE. Using patient samples with threshold concentrations of specific IgE (0.35–0.5 kU/L), the estimated detection limit was 5.1 ± 0.6 ng/µL. This limit was more than 100-fold lower than the total protein concentration typically found in food extracts provided by manufacturers for diagnostic or therapeutic applications.

**Figure 2:**
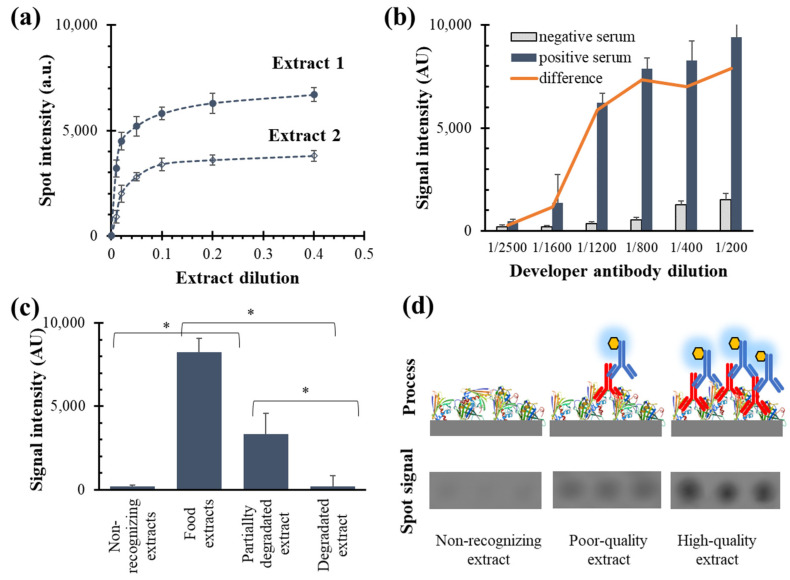
Spot intensities depended on extract dilution (**a**), developer antibody dilution (**b**), extract status (**c**), and extract quality (**d**). Tested target allergy: cow’s milk. Replicates = 3 spots × 3 samples (mean ± standard deviation). Two-tailed *t*-student tests (*p*-value): * indicates lower than 0.001.

The next challenge was to demonstrate that this approach was also valid for protein isolates or concentrates derived from natural sources. Experiments were performed to confirm the proper binding of allergenic molecules to the chip surface without compromising their biological activity. In this study, a direct attachment was approached based on the native functional groups of proteins. The selected strategy was chemisorption based on a hydrophobic bonding between the polycarbonate surface and non-polar amino acid residues, such as alanine, valine, leucine, isoleucine, phenylalanine, tyrosine, tryptophan, proline, and methionine. The incubation of protein solutions in carbonate buffer promoted their passive attachment to a plastic surface. This buffer maintains an optimal pH (typically around 9.0–9.5) and an alkaline environment that enhances binding efficiency by promoting stronger electrostatic interactions and hydrophobic bonding with the plastic surface. The optimal concentration of the coated extract was optimized by checkboard titration assays (Figure 2a). Low concentrations result in poor signal-to-noise ratios and may lead to weak or unstable attachment. Conversely, an excessive amount may cause non-specific binding or aggregation, interfering with specific interactions and compromising assay specificity. The relative standard deviation values remained below 15% for all dilutions, but better responses were achieved for dilution factors between 1/10 and 1/20 depending on the extract or manufacturer. The extract volume required to assess its biological activity was also excellent, as only 40 nL per spot was needed. Therefore, we proved that the method could reliably estimate the biorecognition capability of a complex mixture of proteins.

The experiment on the developer antibody revealed a response curve where the signal for the IgE-containing solutions increased with reagent concentration until it reached a saturation point (Figure 2b). Maximum signal generation suggested that the antibody concentration was sufficient to occupy available binding sites fully. In contrast, the blank and negative samples maintained a constant low signal, demonstrating minimal non-specific binding or background noise. This clear distinction between the positive and negative responses highlights the assay selectivity. The results indicated that the proposed method is an excellent strategy that combines single-molecular docking and a dot-blot platform.

Protein extracts were also subjected to levels of heat exposure to assess their stability and impact on biological activity (Figure 2c). As the thermal treatment intensity increased, the assay response progressively decreased. The spot intensity was null for the most aggressive treatment, indicating a total loss of biorecognition activity. This observation suggests that severe heat exposure can significantly impair or destroy functional integrity. Under the selected working conditions, the array spot image was correlated with biological activity against the allergy biomarker, demonstrating a clear relationship between signal intensity and extract quality (Figure 2d). Extracts obtained from non-allergenic foods produced no visible spots. Conversely, high-quality fractions generated strong signal intensities, aligning with their enhanced recognition capability.

Briefly, these results confirmed that our approach aligns with the ELISA test in assessing the biological activity of a single food extract [9,10,11]. However, the protein array method offers a relevant advantage over ELISA, requiring smaller volumes of extract (about 40 nL/droplet against 100 µL/well), making them more suitable for comprehensive and parallel screening.

### 3.3. High-Throughput Assay Performances

**Figure 3:**
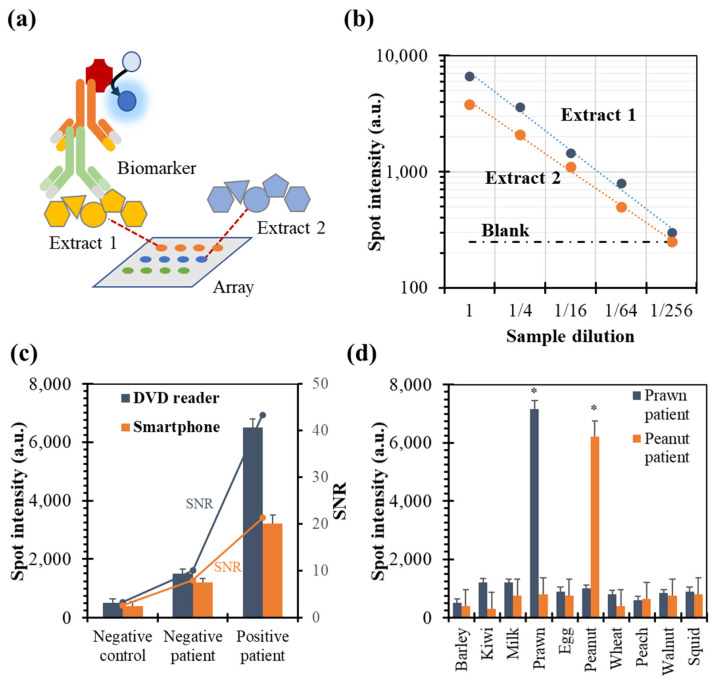
Optimization of multiplex assay conditions. Spot intensities depend on extract dilution before spotting (**a**), sample dilution (**b**), detection technology (**c**), and cross reactivity (**d**) evaluated analyzing patient serum suffering from several allergies. Tested target allergies: cow’s milk, prawn, and peanut. Replicates = 12 (4 spots × 3 samples). Spot intensity: mean ± standard deviation. SNR: signal-to-noise ratio. Two-tailed *t*-student tests (*p*-value): * indicates lower than 0.001.

The protein array method features a unique capability compared to other immunoassay formats by enabling the simultaneous evaluation of multiple extracts or conditions on a single platform, thereby significantly enhancing throughput and operational efficiency. An assay that determined numerous food extracts in the same array was developed, demonstrating this hypothesis. The products were chosen considering the frequencies of allergen reactions in Spain, which mainly correspond to the frequencies in South and Central Europe. The allergens included ten species: barley, kiwi, milk, prawn, egg, peanut, wheat, peach, walnut, and squid. Reagents were applied in an assay format onto the chip (Figure 3a), and standard solutions containing specific IgE from patients were subsequently dispensed. An array image was obtained, and signals from different spot coordinates were analyzed.

Regarding the unspecific recognition, negative responses were observed when the assay was conducted with a pooled serum from non-allergic patients (*n* = 15). Thus, the absence of interference from other serum components on all immobilized extracts was confirmed (Figure 3b). After incubation with serum from allergic patients, detectable spots were captured as expected. However, in a multiplex assay, a possible problem is the lack of linearity, leading to the inaccurate or unreliable quantification of biorecognition level or protein concentration [26]. This deviation can compromise the reproducibility and validity of experimental results, affecting data interpretation and downstream analyses. A serial dilution assay was performed using a pool of samples from positive patients (n = 7) in control blood serum (negative) to check the correct quantification. For example, Figure 3b shows data from the linearity of dilution assay for the two tested products from two manufacturers. The linear response was excellent across a broad range of dilutions, indicating that the methodology offered flexibility in testing serum samples with varying levels of specific IgE. The detection limit and working concentration range of the assay are well-suited to the reported levels of specific IgE (0.1–0.35 kU/L), ensuring accurate and reliable measurements within clinically relevant thresholds [6,26]. As depicted in the figure, differences in signal intensity were observed among manufacturers, but the lines were parallel. The t-test confirmed that the slopes (1: 0.998 ± 0.041; 2: 0.948 ± 0.068) were comparable, with a *p*-value = 0.246.

The developed assay was performed on a polymeric plastic surface, using an array developer that generates a colored product (blue-violet precipitate) and a spot diameter of 450 ± 30 nm. This strategy was compatible with low-cost detectors to image the microarray, eliminating the need for chemically modified glass chips and complex fluorescence measurement devices. In this study, images of protein microarrays obtained using a DVD reader and a smartphone were compared as examples of two optical reading principles, i.e., scanning and camera capturing, respectively (Figure 3c). For both non-fluorescence technologies, the responses for allergic patients were reproducible (deviation lower than 10%) and statistically higher than blanks and serum obtained for non-allergic individuals. Both hand-held optoelectronic devices have the potential to make biosensing more accessible to society [27]. However, the signal and the signal-to-noise ratio were superior for the DVD reader. Therefore, this more sensitive device adapted for bioanalytical purposes is recommended for a better clinical diagnosis or discrimination of extracts depending on their quality.

Concerning assay selectivity in a simultaneous determination, correct discrimination is challenging due to numerous homologous, cross-reactive proteins found in edible foods and aeroallergens [28,29]. A cross-reactivity study analyzed serum obtained from patients suffering from other food allergies, employing a disc with arrays of ten extracts (three replicates), as shown in Figure S4. The aim was to evaluate potential unintended interactions between related IgE and the selected extracts. The spot responses discerned the absence of cross-reactivity (Figure 3d) because no unspecific signals were observed for the remaining proteins. Only visible spots, i.e., signals higher than the blanks, were observed when the extracts corresponded to the allergenic biomolecules.

One limitation of this assay, as well as ELISA and other immunoassays for allergy diagnosis, is the need for operator intervention to perform tasks such as dispensing reagents or washing the surface. As recently published, miniaturization and automation with microfluidic systems can address this issue [30]. Currently, the developed protein array technology is already a valid and effective system, enabling researchers and manufacturers to conduct high-throughput, multiplexed analyses, providing a comprehensive view of recognition patterns or biological activity.

### 3.4. Comparison of Manufacturers

**Figure 4:**
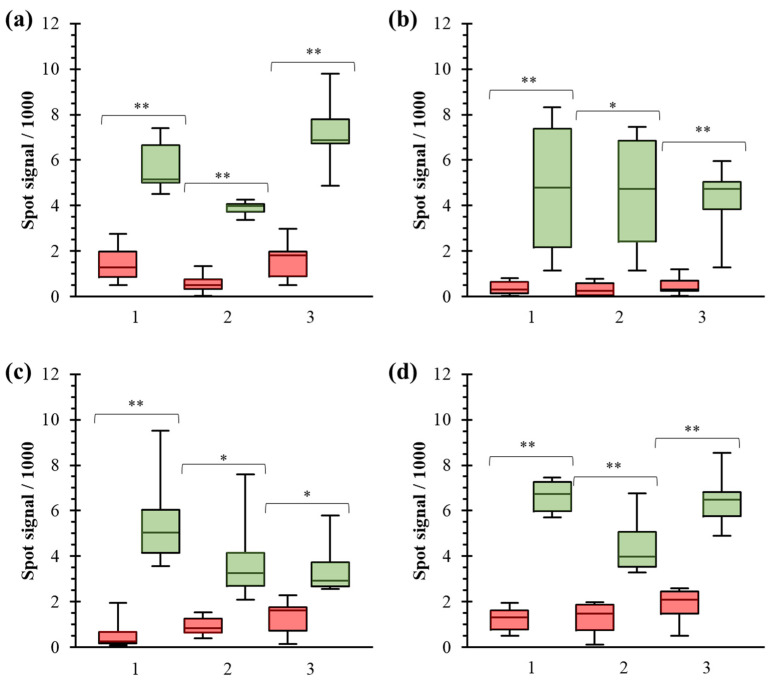
Optical responses registered depending on the allergen extract and classified as a function of the patient group: negative (red) and allergic (green). (**a**) barley, (**b**) kiwi, (**c**) cow’s milk, (**d**) prawn. Two-tailed *t*-student tests (*p*-value): * indicates lower than 0.01, and ** indicates lower than 0.001.

The method was applied to studying several commercial pharmaceutical products to diagnose the most common food allergies. The extracts were immobilized onto the chip surface in an array format: 3 (manufacturers) × 4 (replicates), as shown in Figure S5. Sera obtained from non-allergic individuals and patients allergic to the respective foods were incubated, and resulting images were captured. All the patients were characterized based on their clinical history and in vivo tests. The representation of the signal intensities from the spots enabled their comparison (Figure 4 and Figure S6). Boxplots and student’s t-tests confirmed that all solutions allowed for accurate discrimination between both populations (t_exp_ > t_table_, *p*-valor < 0.01, Table S3). This non-overlapping distribution suggests the assay has strong discriminatory power, enhancing its clinical sensitivity and specificity for detecting food allergies. Therefore, the rate of false positive/negative was zero for all commercial products studied. However, the recorded signal varied significantly depending on the extract origin in four foods (Tables S4 and S5).

Differences between manufacturers were identified using statical tests for barley, prawn, egg, and peanut. This pattern highlights the importance of selecting the appropriate extract that best captures the underlying patterns of specific IgE and the potential interference of serum components.

**Figure 5:**
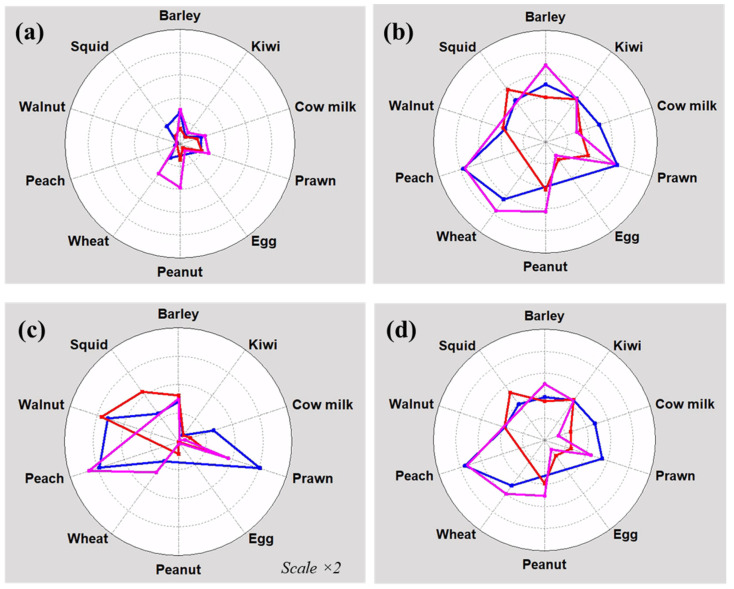
Spider graphs for comparing extracts from different manufacturers: (**a**) Threshold for negatives (Q_4,neg_), (**b**) Threshold for positives (Q_0,pos_), (**c**) Gap between negative and positive responses (Q_0,pos_–Q_4,neg_), (**d**) Mean differences between both populations (Q_2,pos_–Q_2,neg_). Software: Statgraphics Centurion 9. Blue: manufacturer 1; red: manufacturer 2; pink: manufacturer 3.

The array data were used to evaluate extract quality by assessing their ability to differentiate between specific IgE groups. Different parameters were calculated to provide insights into the variability and consistency of test results, examining their potential use for improving industrial practices for their production (Figure 5). Observing the threshold values, certain extracts responded higher than blanks, such as barley, peanut, and wheat. These intensities indicated the presence of an unspecific interaction of serum components or cross-reactivity. It underscores the need for the careful consideration and validation of the production process to ensure robust and reliable diagnosis, avoiding false positives. On the other hand, the higher and minor population gaps were obtained for extracts from prawn, peach, and walnut (2500–3500 a.u.) and t from egg, peanut, and kiwi (40–300 a.u.), respectively. Regarding the origin, differences between manufacturers for barley, prawn, eggs, and peanut were also confirmed. These indicators can serve as valuable tools for assessing the future diagnostic performance of the protein extract because a clear threshold accurately identifies food allergy patients based on the recognition of specific IgE.

These graphs demonstrated that our developed method is feasible for standardizing complicated biological products. Measuring the immunological activity of an extract composed of multiple proteins is particularly challenging, especially when the active ingredients are not well-defined in their nature and composition [11]. Having numerical data makes it easier to quantitatively compare and assess the quality and consistency of allergen concentrates and isolates across different batches and manufacturers [7]. Some assays that rely on separation techniques provide only information about the protein profiles between different pharmaceutical products. However, these results can be insufficient in a clinical setting due to individual variability. In contrast, methods based on IgE biorecognition, like our assay, offer quantitative insights into biological activity using serum, emphasizing its diagnostic power and capability to discriminate between patients and healthy individuals. It also meets these requirements with a minimal consumption of reagents and utilizes affordable instrumentation.

### 3.5. Quality Control Application

Based on the reported results, we thoroughly evaluated the potential for incorporating this technology into the quality systems of the pharmaceutical industry. The identified demand for this type of technology was significant because the development and manufacture of pharmaceutical products from biological sources are complex, expensive, and time-consuming. Thus, more robust and rapid methods to screen and validate them are urgently needed to enhance production efficiency and to ensure their reliability in clinical application. Concerning competitive analysis, advancements in robotics for quality control, particularly those utilizing automatic liquid dispensation and reagent handling, ensure greater consistency than manual operations. However, implementing high-performance bioanalytical assays leads to cost-effective improvements for massive screening.

On the other hand, evaluating allergen extracts entails a multidimensional process, including assessing their biological utility, purity, and stability. Serological methods relying on the detection of allergen-specific IgE have risen to prominence as the primary approach in the pharmaceutical industry and allergy research. However, the application of omics technologies for studying allergens is still an emerging discipline aimed at analyzing numerous extracts in parallel [31]. Building on the strategies used in drug discovery [32], we propose an experimentation tool for the versatile, robust analysis of intermediate and final products. A better-quality assessment can help maintain product integrity, optimize performance, and meet regulatory standards, ultimately enhancing their effectiveness and safety.

## 4. Discussion

**Figure 6:**
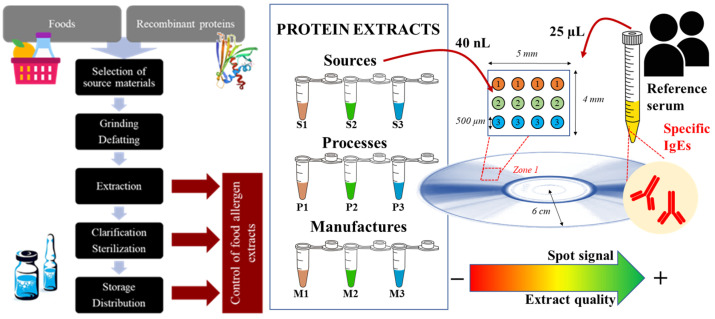
Integration of novel technology in manufacturing food allergen extracts for pharmaceutical applications. Red dot square represents the disc zone for analyzing reference sample.

In practice, the novel technology enables the high-throughput capabilities demanded for a rapid screening tool [33]. The potential impact of this protein array detection method on clinical diagnosis has been previously demonstrated [25,30]. Specific devices allowed for the portable, accurate, sensitive, multiplexed detection of specific IgE in serum, overcoming the traditional prick tests and in vitro methods. In this study, a device was developed to support decision making in the pharmaceutical industry and research area by assisting the obtention of the biological products required as regents for diagnostic tests or immunotherapy (Figure 6). 

The key features and limitations of this novel protein array technology were identified. The device developed is based on a platform with high fabrication quality and cost-effectiveness, such as a DVD disc and DVD-based reader, enabling the high-throughput analysis of biorecognition events. Its affordability makes it accessible for diverse applications, while its precise signal capture ensures the accurate differentiation of extract quality. Nevertheless, the current implementation can be improved. The method relies on an expensive dispensation setup for immobilizing the protein extract, which could limit widespread adoption. The alternative, lower-cost dispensing methods, such as stamping or low-cost pin-based systems, could significantly reduce costs without compromising performance. Furthermore, only a small area of the DVD surface was utilized for sample analysis, but this limitation is not due to issues with dispensation or detection capabilities. The challenge lies in preventing sample cross-contamination, which is typically addressed by creating compartments using chambers or hydrophobic barriers [30]. Given the low cost of the platform, it is often more efficient to use separate arrays with fewer samples per disc [23,24]. For instance, by incorporating additional radii at 3.5 cm and 5.5 cm, the capacity could be expanded to 60 protein arrays per disc without requiring compartmentalization.

Regarding the applications and its performance in quality evaluation, the novel device can be used to differentiate protein extracts from foods obtained from different sources, evaluate the effectiveness of production processes, or compare different manufacturers, as demonstrated in this study. For instance, the complexity and variability inherent in natural sources underscore the importance of robust and efficient screening methods to ensure consistency and quality throughout their production [14]. Our findings indicate that this new method offers a faster, more precise, and sensitive measure of allergenic activity compared to traditional approaches like immunoblotting or ELISA (Table 1). Additionally, arrays save cost and reduce reagent consumption through miniaturized approaches. The volume is minimal, with many functions integrated into a chip spanning several centimeters.

Another distinctive feature of our assay is its quantitative nature. Differentiating the quality levels is crucial for ensuring consistency and reliability in downstream applications, such as diagnostic assays or therapeutic formulations. The outcome of our method (methodology and device) can be a set of quality indicators obtained from the spot intensities. These indicators can serve as a direct estimation of the effectiveness of immunotherapy, the risk of adverse reactions, or the specificity of diagnostic tests. In fact, the gap size between patient populations can be related to the Youden cut-off. This index is a metric that combines sensitivity and specificity into a single value and is often used to determine an optimal cut-off point for diagnostic tests and, consequently, to determine the potential false positives and false negatives.

## 5. Conclusions

The developed technology is profitable for the massive monitoring of the manufacturing steps of food-derived products, as it relies on its genuine biological capability to interact with specific IgE. The conclusion was that the reverse-phase allergen array used protein extract in microspot format. As part of a minimal consumption of reagents, the analytical performance was outstanding, characterized by high selectivity, excellent repeatability, and low detection limits.

Our solution offers advantages over other approaches applied in the quality control of protein extracts. (i) The arrays enable parallel analyses of several reactions to identify effective proteins for this particular molecular process, thanks to the rapid progress of high-throughput screening. (ii) Its versatility is demonstrated by its ability to facilitate comparisons among different sources, intermediate products, or manufacturers. (iii) The technology provides quantitative information essential for ensuring batch-to-batch consistency, as mandated by regulatory requirements. (iv) The analytical and operational performances of the method based on polycarbonate chip/smartphone or DVD reader overcome previous approaches based on glass chips/fluorescence scanner or ELISA plate/reader. The main limitation is the availability of representative immunoglobulin standards or patient sera. However, this drawback is common in most in vitro techniques.

The potential users of the proposed quality control test are diagnostic and therapeutic companies mainly focusing on protein purification, allergies, and intolerances. The candidate pharmaceutical products include protein mixtures concentrated or isolated from foods, pollen, and other aeroallergens. These extracts are used in various clinical applications, such as skin prick, patch, and IgE blood tests, to identify immune responses to specific proteins. The approach may also be utilized in research settings to study the mechanisms of allergies and develop new diagnostic devices, such as wearable biosensors. Overall, the new method can contribute to the primary goal of accurately identifying and managing immunoglobulin-mediated allergy.

## Figures and Tables

**Table 1:** Comparison of techniques for assessing the biological activity of protein extracts applied in the pharmaceutical industry and allergy research.

Techniques	Nature	Advantages	Drawbacks
Skin test	In vivo	Real information about reactivity	Ethics restriction, discomfort for individuals, risk of adverse reactions.
Basophil activation test	Ex vivo	Valuable insights into the allergic mechanism	Complexity, high cost, more time consumption, requirement of specialized equipment and expertise, and limited standardization across laboratories
ELISA	In vitro	Possible automation, high sensitivity, specificity, and ability to quantify accurately	Limited working capability, specialized equipment and reagents
Electrophoretic techniques	In vitro	Easy visualization of protein profile	Limited information about reactivity, qualitative data, low working capability
Array technology	In vitro	Miniaturization, multiplexing, quantitative data, high throughput, low consumption of extract	Needs correct immobilization

## Data Availability

Data is contained within the article or Supplementary Materials.

## Supplementary Materials

The following supporting information can be downloaded at: https://www.mdpi.com/article/10.3390/bios14120615/s1, Figure S1. Smartphone sensing; Figure S2. DVD sensing; Figure S3. Signal intensity as a proxy for protein extract quality assessment; Figure S4. Cross-reactivity study; Figure S5. Array images captured from human samples using reagents supplied by different manufacturers.; Figure S6. Optical responses registered depending on the allergen extract and classified as function of patient group. Table S1. List of tested food extracts; Table S2. Main features of polycarbonate chips; Table S3. Comparative results for negative and positive patients utilizing the proposed assay, stratified by manufacturer extract; Table S4. ANOVA multifactor and test of least significant difference (LSD); Table S5. Levene test, test of least significant difference (LSD), ANOVA, and Kruskal-Wallis test (KW) [34,35,36,37,38,39,40,41,42,43,44,45,46,47,48,49,50,51,52,53,54].
