# Supplementary material for: Massive Screening of Food Extracts for Quality Assessment and Standardization of Allergenic Activity
Source: Biosensors (Basel). 2024 Dec 13;14(12):615. doi: 10.3390/bios14120615 (PMC11674539; doi:10.3390/bios14120615)
Supplement: Supplementary file 1 [file biosensors-14-00615-s001.zip › biosensors-3315234-supplementary.pdf]

Massive Screening of Food Extracts for Quality Assessment and Standardization of Allergenic Activity

Luis A. Tortajada-Genaro [1,2,3]

[1] Instituto Interuniversitario de Investigación de Reconocimiento Molecular y Desarrollo Tecnológico (IDM), Universitat Politècnica de València, Universitat de València, Camino de Vera s/n, E46022 Valencia, Spain; luitorge@qim.upv.es; Tel.: +34-963877000
[2] Departamento de Química, Universitat Politècnica de València, Camino de Vera s/n, E46022 Valencia, Spain
[3] Unidad Mixta UPV-La Fe, Nanomedicine and Sensors, Instituto de Investigación Sanitaria La Fe, Valencia, Spain

SUPPLEMENTARY MATERIAL

TABLES

Table S1. List of tested food extracts.
Table S2. Main features of polycarbonate chips.
Table S3. Comparative results of T-student test for negative and positive patients utilizing the proposed assay, stratified by manufacturer extract.
Table S4. ANOVA multifactor and test of least significant difference (LSD).
Table S5. Levene test, test of least significant difference (LSD), ANOVA, and Kruskal-Wallis test (KW)

FIGURES

Figure S1. Smartphone sensing.
Figure S2. DVD sensing.
Figure S3. Signal intensity as a proxy for protein extract quality assessment.
Figure S4. Cross-reactivity study.
Figure S5. Array images captured from human samples using reagents supplied by different manufacturers.
Figure S6. Optical responses registered depending on the allergen extract and classified as function of patient group.

Table S1. List of tested food extracts. *Manufacturer code (M1, M2, and M3) and described allergen proteins contained in the food extracts.*

	Allergen	Scientific name	M1	M2	M3	Common allergen proteins
1	Barley	*Hordeum vulgare*	x	x	x	Lipid Transfer Protein<br>Alpha-amylase inhibitor<br>Beta-amylase
2	Cow's milk	*Bos taurus*	x	x	x	Casein<br>β-Lactoglobulin<br>α-Lactalbumin
3	Kiwi	*Actinidia deliciosa*	x	x	x	Actinidin<br>Thaumatin-like protein
4	Prawn	*Penaeus monodon**<br>*Litopenaeus vannamei**	x	x	x	Tropomyosin<br>Arginine kinase
5	Chicken egg	*Gallus gallus domesticus*		x	x	Ovomucoid<br>Ovalbumin<br>Ovotransferrin<br>Lysozyme
6	Peanut	*Arachis hypogaea*		x	x	storage proteins such as Ara h 1,<br>Ara h 2, Ara h 3, Ara h 6
7	Wheat	*Triticum aestivum*	x		x	ω-5 Gliadin<br>Glutenin<br>α-Gliadin
8	Peach	*Prunus persica*	x		x	Lipid Transfer Protein<br>Profilin
9	Walnut	*Juglans regia*	x	x		2S albumin (Jug r 1)<br>Non-specific lipid transfer protein (Jug r 3)
10	Squid	*Loligo vulgaris**	x	x		Tropomyosin<br>Myosin light chain

* Multiple species for the same common name

Table S2. Main features of polycarbonate chips. *Advantages of polycarbonate as substrate for chips in quality control, diagnostic, and research applications.*

**Feature**	**Advantage**	**Impact**
Cost-Effectiveness	Affordable and widely available material	Reduces overall costs for large-scale manufacturing and experimental setups
Mechanical Strength	High toughness, impact resistance, and rigidity	Ensures durability and reliability in handling and experimental conditions
Heat Resistance	Maintains integrity under elevated temperatures	Suitable for processes requiring heat, such as sterilization or thermal treatments
Optical Properties	Transparent and compatible with optical detection methods	Facilitates efficient signal readouts in imaging and optical-based sensing
Moldability	Easily shaped through injection molding	Enables cost-effective production of complex designs, including microfluidic architectures
Surface Chemistry	High immobilization yield via adsorption or after surface activation	Enhances probe attachment efficiency, improving assay sensitivity and reproducibility
Hydrophobic Nature	Reduces non-specific binding of sample components	Improves assay specificity and accuracy

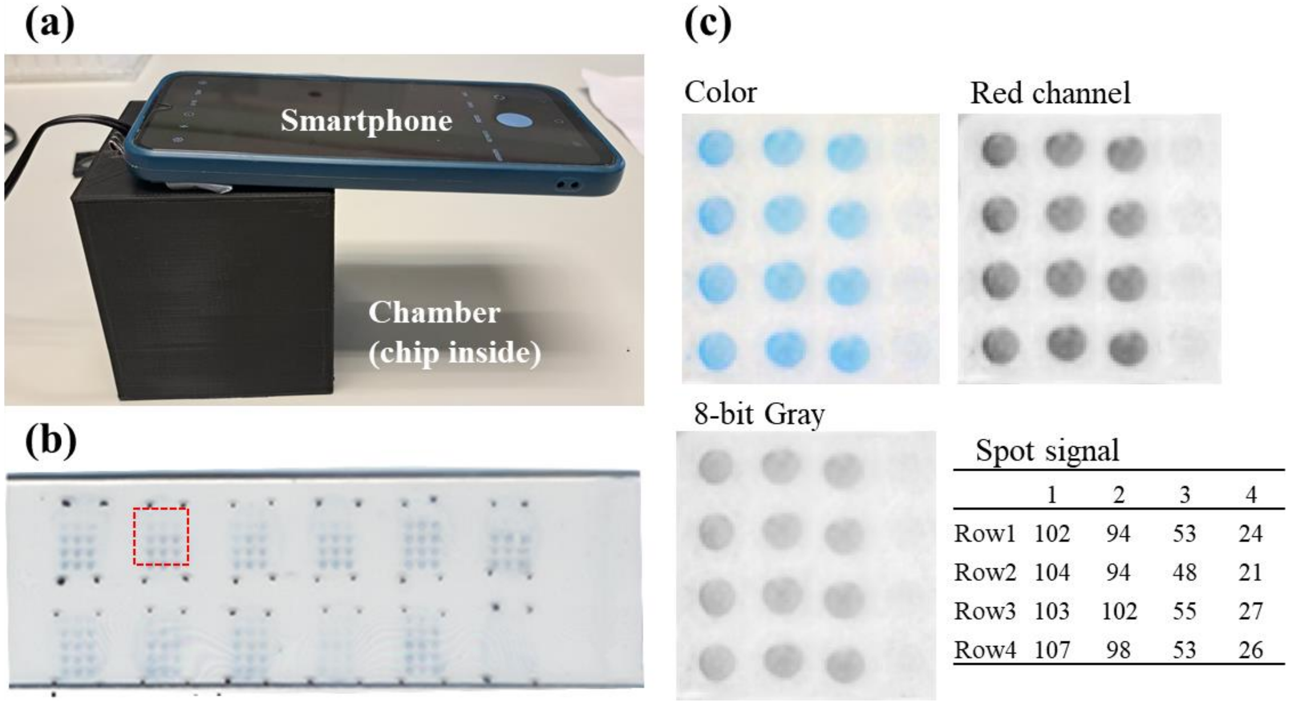

Figure S1. Smartphone sensing. *(a) Setup for image capturing by smartphone camera. Smartphone: Samsung Galaxy A15 5G. Camera: 50 MP (f/1.8). Dimensions: 160.1 x 76.8 x 8.4 mm. Weight: 200 g. The chip was placed inside a dark chamber illuminated by diffuse light to ensure even lighting and minimize reflections. The distance between the camera and the chip was optimized to obtain a clear, reproducible, and focused image. (b) The image shows a planar chip containing several arrays on its surface, with each array corresponding to a different sample: 12 samples per chip. (c) Image processing and data output. Using the free-software ImageJ, the image of 4×4 array was split into its RGB channels. For red channel, intensity profiles were generated for the pixels corresponding to each spot on the chip, yielding the associated spot intensity.*

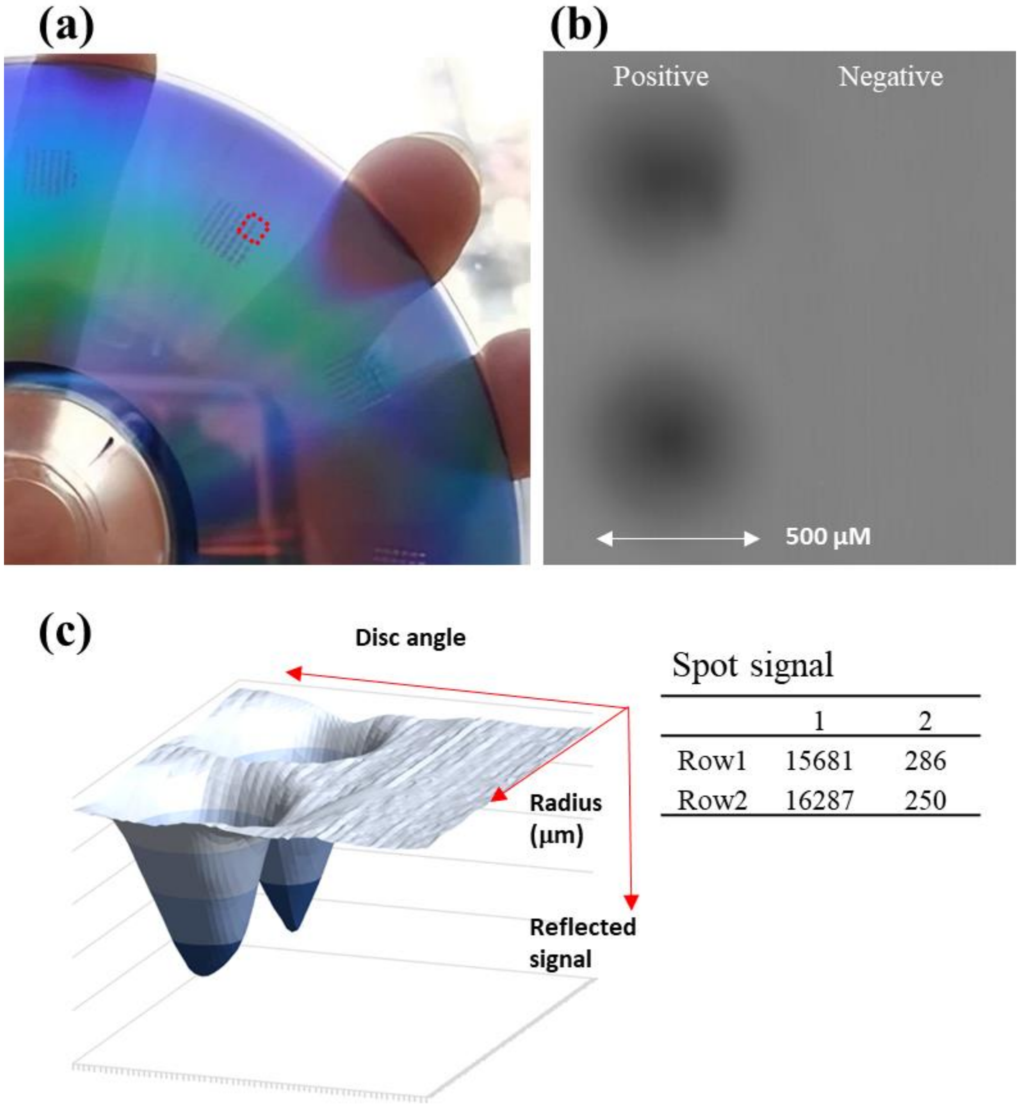

Figure S2. DVD sensing. *(a) The image shows a disc containing several arrays on its surface, with each array corresponding to a different sample (20 samples per disc). (b) A fragment of the captured image from the DVD reader shows two positive spots (allergen extract) and two negative spots (non-allergen extract). Reader resolution: 1 pixel = 10 µm. (c) Image processing and data output. The figure represents the signal intensity for each pixel, defined by its radius and angle. The spot signal is obtained from the pixel intensities associated with that spot.*

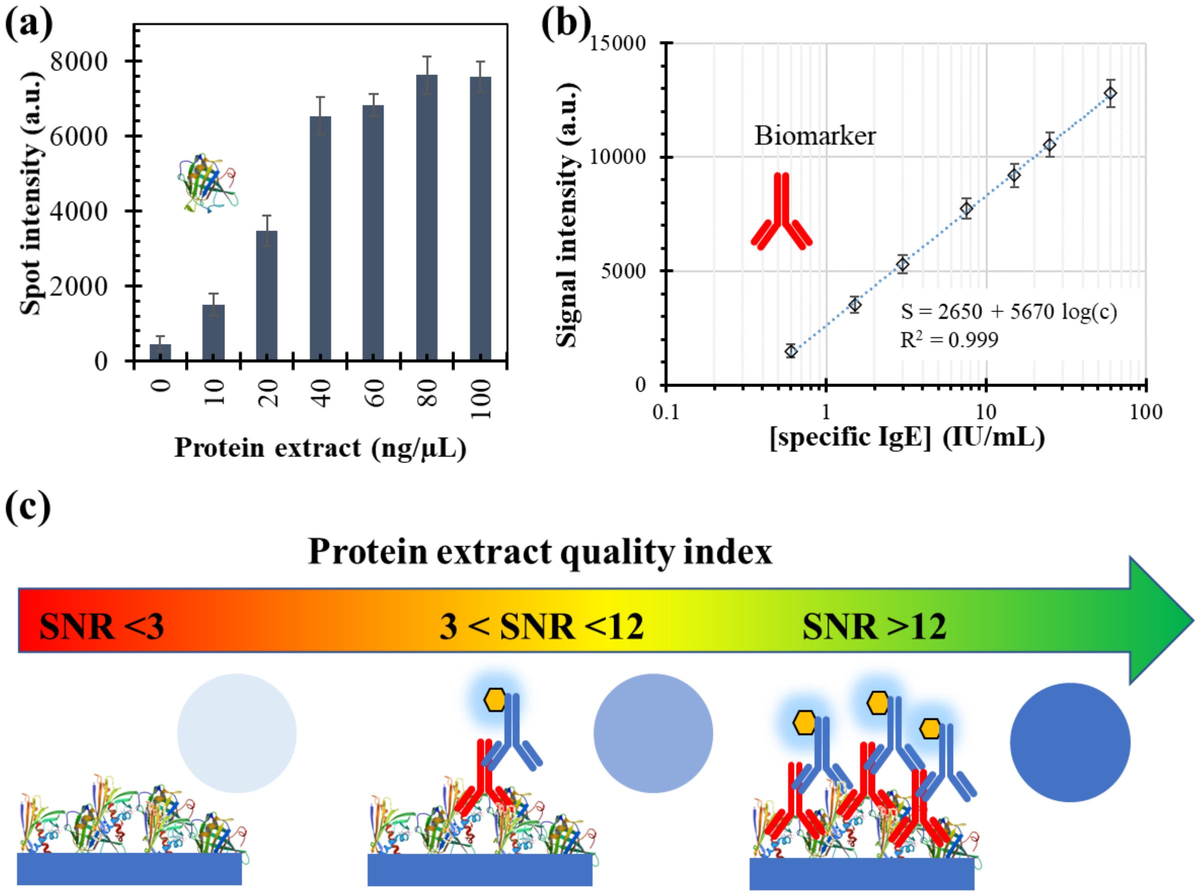

Figure S3. Signal intensity as a proxy for protein extract quality assessment. *Spot intensities depended on: (a) amount of protein extract and (b) amount of specific IgE. (c) The quality index of a protein extract, as determined by its biological activity, can be directly correlated with the signal-to-noise ratio (SNR) obtained from the microarray assay using a determined amount of specific IgE: (left) extract with null recognition capability; (central) extract with low recognition capability; (right) extract with high recognition capability. Replicates = 3. Specific IgE were quantified by ImmunoCAP technology. The detection limit, defined as the lowest extract concentration that can be reliably determined, was calculated from blank measurements. The estimated value was 5.1 ± 0.6 ng/µL for protein extract.*

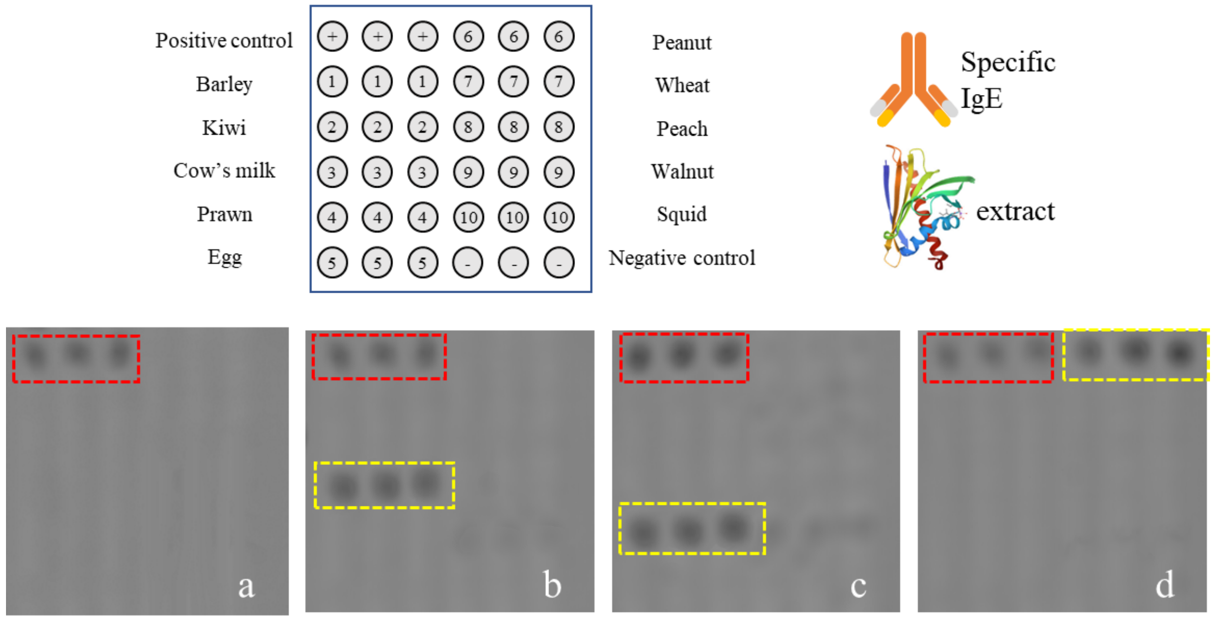

Figure S4. Cross-reactivity study. *(Top) Scheme of array layout for the simultaneous assessment of ten protein extracts and assay controls. (Bottom) Examples of array images obtained for a serum sample from the recruited individuals. (a) non-allergic; (b) milk allergy; (c) prawn allergy; (d) peanut allergy.*

Extract	Negative patient	Positive patient
**Barley**	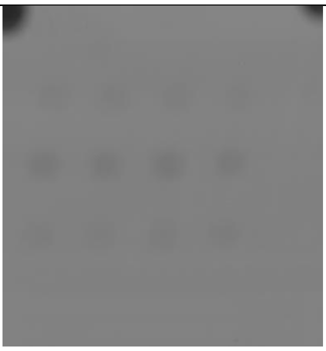	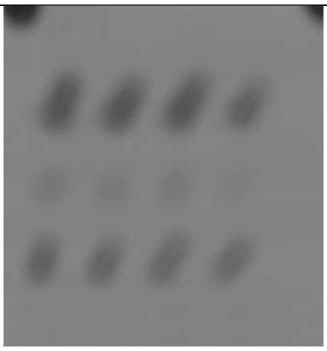
**Kiwi**	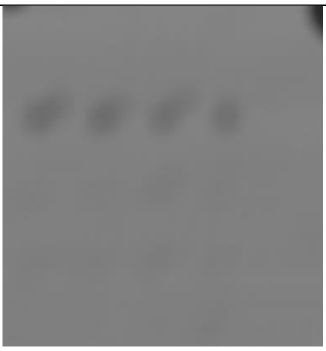	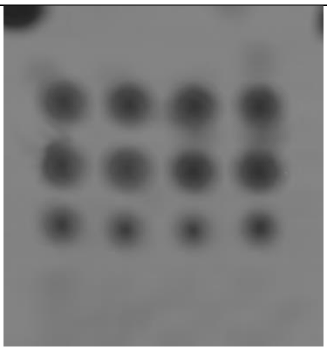
**Cow's milk**	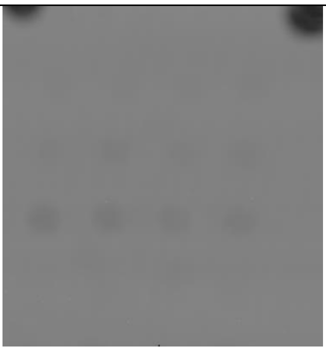	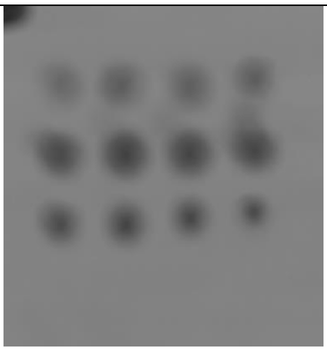
**Prawn**	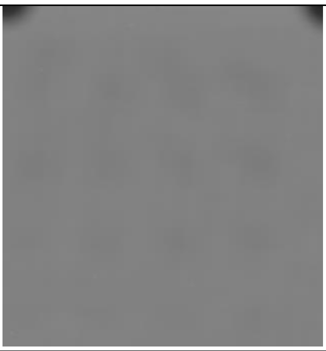	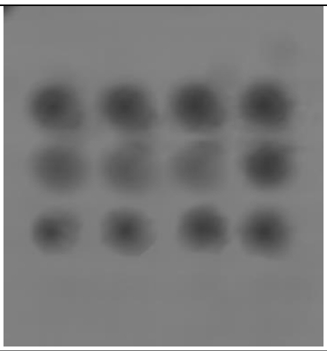

Figure S5. Array images captured from human samples using reagents supplied by different manufacturers. *Patient group was assigned based on the clinical history and the concentration of specific IgE quantified by ImmunoCAP technology. Array format is 3x4, including 12 proteins extracts distributed in manufacturers (rows) and replicates (columns). The patients were diagnosed following the procedure described in the European Network of Drug Allergy (ENDA) protocol based on skin testing and in vitro tests.*

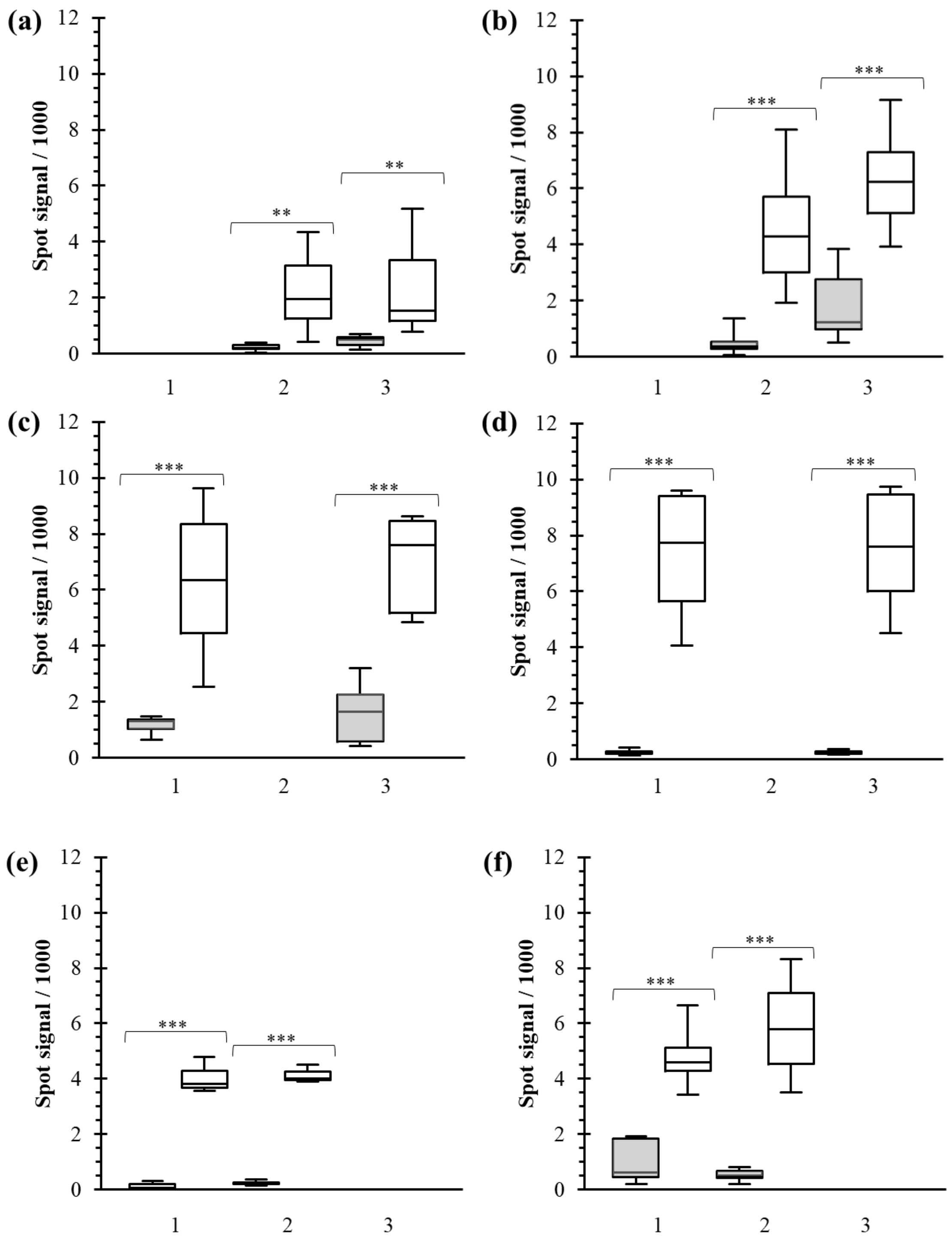

Figure S6. Optical responses registered depending on the allergen extract and classified as function of patient group. *Extract: (a) chicken egg, (b) peanut, (c) wheat, (d) peach, (e) walnut, (f) squid. Patient: negative (grey) and allergic (white). Two-tailed t-student tests (p-value): ** indicates lower than 0.01 and *** indicates lower than 0.001. The patients were diagnosed following the procedure described in the European Network of Drug Allergy (ENDA) protocol based on skin testing and in vitro tests.*

Table S3. Comparative results for negative and positive patients utilizing the proposed assay, stratified by manufacturer extract. *P-values were calculated using a two-tailed Student's t-test. Red values indicate significant difference.*

Manufacturer		Barley	Kiwi	Cow milk	Prawn	Egg	Peanut	Wheat	Peach	Walnut	Squid
M1	t	8.142	3.453	5.826	14.797			4.770	8.375	16.776	6.004
	p-value	0.001	0.001	0.000	0.000			0.001	0.000	0.000	0.000
M2	t	16.241	3.777	3.801	5.470	3.659	4.840			33.076	6.921
	p-value	0.000	0.004	0.003	0.000	0.003	0.000			0.000	0.000
M3	t	8.414	4.839	3.831	8.196	2.890	5.449	6.464	9.227		
	p-value	0.000	0.001	0.003	0.000	0.014	0.000	0.000	0.000		

Table S4. ANOVA multifactor and test of least significant difference (LSD). *Red values indicate significant difference.*

Variable		Barley	Kiwi	Cow milk	Prawn	Egg	Peanut	Wheat	Peach	Walnut	Squid
Manuf.	F	15.22	0.02	0.72	5.42	0.19	7.63	0.61	0.03	0.63	0.01
	p-value	0.000	0.977	0.495	0.009	0.067	0.011	0.445	0.875	0.439	0.930
Patient	F	184.96	46.81	53.07	182.42	21.63	54.79	62.67	160.43	949.87	78.11
	p-value	0.000	0.000	0.000	0.000	0.000	0.000	0.000	0.000	0.000	0.000
LSD	groups	M2, M1-M3			M2, M1-M3		M2, M3				

LSD test helps to identify the populations whose means are statistically different.

Table S5. Levene test, test of least significant difference (LSD), ANOVA, and Kruskal-Wallis test (KW).

Patient group: negatives (a) and positive (b). Red values indicate significant difference.

(a)

Test		Barley	Kiwi	Cow milk	Prawn	Egg	Peanut	Wheat	Peach	Walnut	Squid
Levene	value	1.724	0.435	1.431	0.544	1.944	12.900	9.711	0.392	4.143	51.373
	p-value	0.207	0.654	0.265	0.590	0.189	0.004	0.009	0.543	0.065	0.000
ANOVA	F	3.66	0.36	2.2	1.91	6.02	7.99	0.84	0	4.05	2.84
	p-value	0.046	0.701	0.139	0.176	0.030	0.015	0.377	0.992	0.067	0.118
LSD	groups	M2, M1-M3				M2, M3	M2, M3				
KW	value	5.194	0.475	4.159	3.534	3.931	6.222	0.2	0.104	2.183	0.696
	p-value	0.075	0.789	0.125	0.171	0.047	0.013	0.655	0.747	0.140	0.404
	groups	M2, M1-M3				M2, M3	M2, M3				

(b)

Test		Barley	Kiwi	Cow milk	Prawn	Egg	Peanut	Wheat	Peach	Walnut	Squid
Levene	value	3.807	2.898	0.493	0.733	0.258	0.291	0.950	0.129	2.504	1.578
	p-value	0.418	0.107	0.620	0.497	0.621	0.600	0.358	0.726	0.189	0.259
ANOVA	F	15.96	0.04	2.19	6.57	0.03	2.78	0.20	0.02	0.04	0.67
	p-value	0.000	0.960	0.147	0.009	0.874	0.122	0.670	0.879	0.849	0.443
LSD	groups	M2, M1-M3			M2, M1-M3						
KW	value	14.726	0.154	4.714	7.170	0.004	2.550	0.273	0.200	0.429	1.333
	p-value	0.001	0.926	0.095	0.028	0.949	0.110	0.602	0.655	0.513	0.248
	groups	M2, M1-M3			M2, M1-M3						

Levene test helps to identify the homogeneity of variance

LSD test helps to identify the populations whose means are statistically different.

Kruskal-Wallis test to identify the populations whose medians are statistically different.
